# Cyclic ST-segment elevation after primary percutaneous coronary intervention

**DOI:** 10.1007/s12471-020-01498-8

**Published:** 2020-10-09

**Authors:** W. P. J. Jansen

**Affiliations:** Department of Cardiology, Tergooi Hospital, Blaricum, The Netherlands

**Fig. 1:**
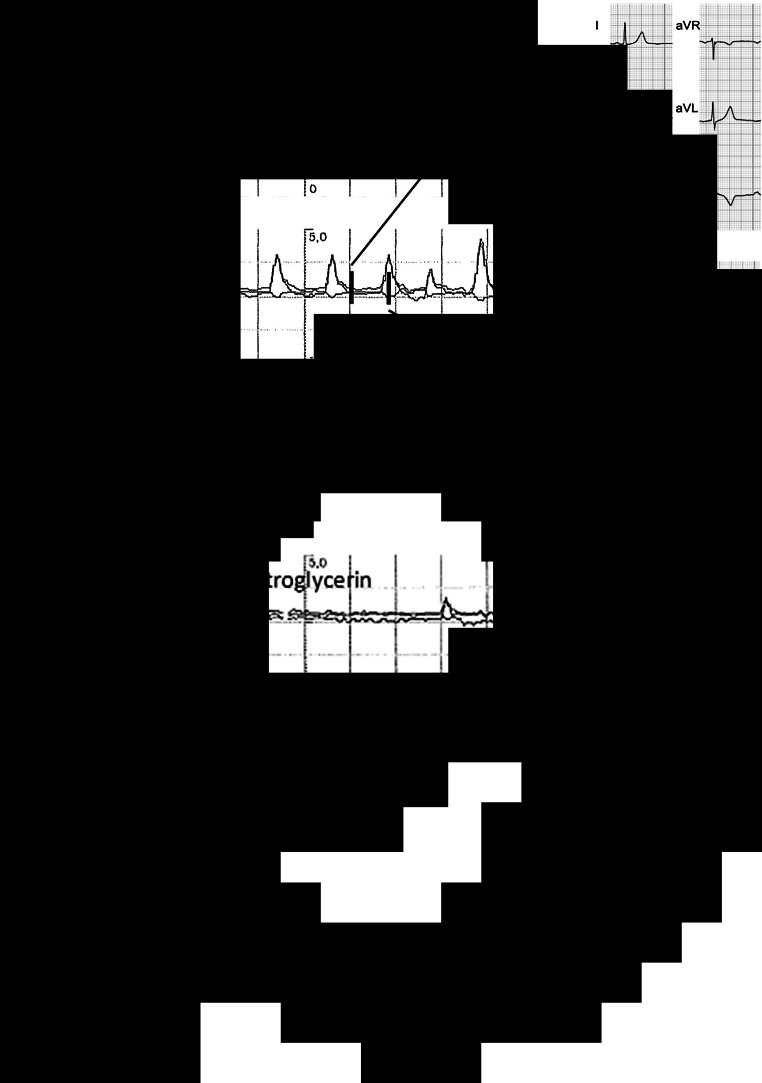
**a** Heart rate (HR) trend and ST-segment trend (*left*) showing repetitive ST-elevation with 14-minute intervals, without concomitant HR variation. Electrocardiography recordings (*right*) confirm ST-elevation in inferior leads. **b** Complete resolution of ST-elevations after application of sublingual nitrate spray, only to reoccur after 1 h, in the same repetitive manner

A 44-year-old man was admitted for an inferior ST-elevation myocardial infarction (STEMI), for which he underwent successful primary percutaneous coronary intervention of the occluded right coronary artery (RCA). Several hours later, ST-segment monitoring revealed a strikingly regular pattern of intermittent inferior ST-elevation, confirmed by 12-lead electrocardiography (Fig. 1a). Following sublingual administration of nitroglycerin, the ST-elevation completely resolved; however, it reoccurred after 1 h (Fig. 1b). Repeat angiography showed a patent RCA with Thrombolysis in Myocardial Infarction (TIMI) III flow. Intravenous nitrates and oral diltiazem were administered, after which the phenomenon did not reoccur.

Cyclic ST-elevation after acute coronary syndromes has been described previously [1]. I found one similar case of intermittent ST-elevation after NSTEMI, which was reversed by intravenous nitrates [2]. Several hypotheses have been postulated, including coronary vasospasm and platelet-mediated thrombus formation [3, 4]. Angiographic results and response to spasmolytic agents suggest a vasospastic phenomenon. The explanation for the strikingly cyclic pattern, however, remains to be elucidated.
